# Gene and drug delivery system and potential treatment into inner ear for protection and regeneration

**DOI:** 10.3389/fphar.2014.00222

**Published:** 2014-10-08

**Authors:** Sho Kanzaki

**Affiliations:** Department of Otorhinolaryngology, Keio UniversityTokyo, Japan

**Keywords:** hair cell, spiral ganglion neuron, protection, regeneration, drug delivery, endoscopy

## Abstract

The most common type of hearing loss results from damage to the cochlea including lost hair cells (HCs) and spiral ganglion neurons (SGNs). In mammals, cochlear HC loss causes irreversible hearing impairment because this type of sensory cell cannot regenerate. The protection from SGN from degeneration has implications for cochlear implant to patients with severe deafness. This review summarizes the several treatments for HC regeneration based on experiments. We discuss how transgene expression of the neurotrophic factor can protect SGN from degeneration and describe potential new therapeutic interventions to reduce hearing loss. We also summarized viral vectors and introduced the gene and drug delivery system for regeneration and protection of cochlear HCs. Finally, we introduce the novel endoscopy we developed for local injection into cochlea.

## POTENTIAL USE OF DRUG AND GENE THERAPY TO AID REGENERATION OF SENSORY CELLS

Drug, gene and cell therapy are attractive to facilitate regeneration of hair cells (HCs) and spiral ganglion neurons (SGNs). For endogenous HC regeneration, it is critical to identify potential stem cells of the inner ear.

We will try to regulate the Notch signaling and overexpress proteins, such as *Atoh 1*, that can increase the regenerative properties of the sensory epithelium of the cochlea (Zheng and Gao, 2000). Adenoviral (ADV) vectors can transfect with SCs *in vitro* (Kanzaki et al., 2002a) and *in vivo* (Ishimoto et al., 2002).

When adenovirus containing *Atoh 1* gene were transfected into SCs through endolymph of the mature guinea pig cochlea, HCs replacement and hearing improvement were observed in some deaf mammals.

The members of the Notch signaling pathway also play key roles in mediating HC differentiation in the developing cochlea (Jahan et al., 2013). HC production is regulated by the Notch signaling pathway in a process called lateral inhibition (Jahan et al., 2013). Notch-mediated lateral inhibition is critical for cell-fate determination within HCs and SCs, regulating the number of HCs by inhibiting HC proliferation.

When Notch signaling is inhibited by a γ-secretase inhibitor, HC differentiation from inner ear stem cells was observed *in vivo* (Mizutari et al., 2013). HC generation resulted from an increase in the level of *Atoh 1* in response to inhibition of Notch signaling (Jahan et al., 2013). HC regeneration resulted from trans-differentiation of SCs (Mizutari et al., 2013). Inhibition of Notch signaling may be a potential therapy to treat severe deafness.

## PROTECTION OF NEURAL DEGENERATION

Following profound hearing loss, severe atrophy is found within the cochlea and central auditory systems. If the peripheral organ is damaged, the nerve degeneration is a common finding in the nervous system (Springer and Kitzman, 1998).

Destruction of IHCs due to trauma or disease leads to the degeneration of SGNs (Otte et al., 1978; Altschuler et al., 1999), probably because IHCs normally provide excitatory activation to the cochlear nerve. Preservation of SGNs, their axons of cochlear nerve and their connections are necessary to restore auditory function, because degeneration of SGNs should reduce the effectiveness of hearing aid devices, including cochlear implant.

Chronic cochlear electrical stimulation (ES) after HC loss has been shown to reduce the irreversible damage of SGNs (Miller et al., 2002).

Neurotrophic factors and maintenance of synaptic connectivity are important to prevent neuronal degeneration (Springer and Kitzman, 1998). Brain-derived neurotrophic factor (BDNF) and/or glial derived neurotrophic factor (GDNF) effectively promote SGN survival following exposure to deafening noise or ototoxic drugs (Miller et al., 2002).

Treatment with neurotrophic factor such as GDNF significantly enhance SGN survival compared to that in untreated deafened ears. Combining ES with GDNF overexpression treatment additionally protects against SGN degeneration (Kanzaki et al., 2002b). It suggests that cochlear implant combined with neurotrophic support may have more effective treatment.

## APPROACHES INTO INNER EAR

The inner ear is enclosed by a bony capsule. Such a relative anatomical isolation makes inner ear an ideal target for therapeutic local injection because spread of the injected vector, the drug, and the transgene to surrounding tissues is expected to be limited.

We have several approaches into inner ear including via scala tympani, scala media, or semicircular canal. Scala media approach would be more effective with cells in organ of Corti, but more traumatic. It means via scala media approach does not preserve hearing threshold. Scala tympani approach is less traumatic and used for prevention of SGN degeneration.

## GENE DELIVERY INTO INNER EAR

Gene delivery or transfer introduces exogenous genes into cells. As mentioned above, viral vectors are still the efficient vehicles for gene transfer in animal experiments about the inner ear regeneration.

For inner ear gene transfer, several types of viral vectors such as ADV, herpes viral (HSV), adeno-associate viral (AAV) (Iizuka et al., 2008), Sendai virus vectors (SEV) (Kanzaki et al., 2007), have been applied to different types of cells of the mammals. However, cytotoxicity and immune response are common complicating factors in the clinical and experimental use of viral vectors.

## DRUG DELIVERY SYSTEM INTO INNER EAR

We describe that treatment for inner ear regeneration and protection needs local injection into round window membrane (RWM) in cochlea, but not systemic injections due to systemic side effects. However, pharmacokinetics of agents locally injected into inner ear is not well known. Hence, we develop the real time observation of drug delivery system in transgenic animals *in vivo* (Kanzaki et al., 2012a). We observed drug delivery time difference between local and systemic injections. In local injection, drugs appeared and disappeared earlier than in systemic injection. However, delivery time also varied in the local injection group.

We also find that RW niche obstruction such as false membrane or fibrous connective membrane blocked drug delivery into inner ear. Therefore, observing RW is very important before local injection of drug into inner ear (Kanzaki et al., 2012a).

## NOVEL ENDOSCOPY FOR DRUG DELIVERY

The RWM cannot be visualized in around 30% of all the patients with hearing loss. In those cases adhesions need to be removed first before local injection is performed. We develop a novel endoscopy to explore the RWM for the purpose of local injection (Kanzaki et al., 2012b). The endoscope we develop contains a catheter channel for delivering drugs and a suction channel. The catheter has a fine needle, which can be used to remove or perforate RW niche mucosal adhesions and be used to apply drugs directly onto the surface of the RWM (Kanzaki et al., 2012b).

## CONCLUSION AND FUTURE DIRECTIONS

The molecular signals that stimulate HCs regeneration and protection of SGNs have begun to be identified. Proliferation of HCs has been achieved in mammals by overexpressing Atoh1, and Notch inhibitor drugs. The preservation of SGNs is very important and has implications for cochlear implants. These studies will lead to therapeutic interventions for the hearing impaired.

## ACKNOWLEDGMENT

This manuscript was supported by funds from Grant in aid (OK 2012–2013) for exploratory research in the Ministry of Education, Culture, Sports, Science, and Technology and grant in aid from the Takeda Science Foundation.

## Conflict of Interest Statement

The author declares that the research was conducted in the absence of any commercial or financial relationships that could be construed as a potential conflict of interest.
